# Experimental data on compressive and flexural strengths of coir fibre reinforced foamed concrete at elevated temperatures

**DOI:** 10.1016/j.dib.2019.104320

**Published:** 2019-07-25

**Authors:** M.A. Othuman Mydin, N. Mohd Zamzani, A.N. Abdul Ghani

**Affiliations:** School of Housing, Building and Planning, Universiti Sains Malaysia, 11800, Penang, Malaysia

**Keywords:** Foamed concrete, Elevated temperature, Coir fibre, Compressive, Flexural, Bending

## Abstract

Quantifying the elevated temperature strengths of cement-based material is crucial to the design of building structural systems for fire resistance purpose. This paper collates a database of elevated temperature axial compressive and flexural strengths of coir fibre reinforced foamed concrete exposed to heating temperatures of 105 °C, 200 °C, 300 °C, 400 °C, 500 °C, 600 °C, 700 °C and 800 °C. There were four densities of foamed concrete of 700, 1100, 1500 and 1900 kg/m^3^ were prepared and tested. The untreated coir fibre was added in foamed concrete in percentages of 0.1%, 0.2%, 0.3%, 0.4%, 0.5%, and 0.6% by mix volume fraction. The database can aid in prediction of elevated temperature strengths of fibre reinforced foamed concrete which can be exploited to assist manufacturers to develop their products without having to perform numerous large-scale elevated temperature tests in the future.

**Table:** Specifications Table

Subject area	*Civil Engineering*
More specific subject area	*Foamed concrete, Coir Fibre, Mechanical Properties, High Temperature Performance*
Type of data	*Tables and Figures*
How data was acquired	*Laboratory experiment and use of relevant standard*
Data format	*Raw and analyzed*
Experimental factors	Heating temperatures of 105 °C, 200 °C, 300 °C, 400 °C, 500 °C, 600 °C, 700 °C and 800 °C
Experimental features	Untreated coir fibre of was added in foamed concrete in percentages of 0.1%, 0.2%, 0.3%, 0.4%, 0.5%, and 0.6% by mix volume. Densities of 700, 1100, 1500 and 1900 kg/m^3^ were cast and tested to determine the elevated temperature mechanical properties such as compressive and flexural strengths. The electric furnace temperature exposure profiles were produced by a programmable microprocessor temperature controller attached to the furnace power supply and monitored by a Type K thermocouple located in the furnace chamber.
Data source location	Penang, Malaysia
Data accessibility	The data are available within this article

**Table:** 

**Value of the data** • This database is beneficial because foamed concrete is a widely used material and relevant information on its uses can be very significant particularly when it is exposed to high temperature and fire condition • The database can assist as an experimental framework for the analysis of other high temperature properties of foamed concrete • The test data allows for exploration on the use of coir fibre as another possible waste material for reinforcement of foamed concrete which tend to be very brittle especially at high temperatures • The database may be relevant in the development of standards or codes of practice for foamed concrete exposed to elevated temperatures • The database presented can be used to develop an optimum method for production of foamed concrete for fire resistance purpose

## 1 Data

This dataset described herein were acquired from the experimental studies conducted to determine the elevated temperature compressive strength and flexural strength of coir fibre reinforced foamed concrete exposed to heating temperatures of 105 °C, 200 °C, 300 °C, 400 °C, 500 °C, 600 °C, 700 °C and 800 °C. Fig. 2, Fig. 4, Fig. 6, Fig. 8 show the compressive strength of 700, 1100, 1500 and 1900 kg/m^3^ density foamed concrete respectively as a function of temperature. Fig. 3, Fig. 5, Fig. 7, Fig. 9 demonstrate the normalized compressive strength of 700, 1100, 1500 and 1900 kg/m^3^ density foamed concrete correspondingly as a function of temperature. In Table 1, Table 2, Table 3, Table 4, percentages of compressive strength retained at each predetermined exposed temperature were summarized for 700, 1100, 1500 and 1900 kg/m^3^ density foamed concrete respectively. Next, Fig. 10, Fig. 12, Fig. 14, Fig. 16 display the flexural strength of 700, 1100, 1500 and 1900 kg/m^3^ density foamed concrete correspondingly as a function of temperature whereas Fig. 11, Fig. 13, Fig. 15, Fig. 17 exhibit the normalized flexural strength of 700, 1100, 1500 and 1900 kg/m^3^ density foamed concrete respectively as a function of temperature. In Table 5, Table 6, Table 7, Table 8, percentages of flexural strength retained at each predetermined exposed temperature were tabulated for 700, 1100, 1500 and 1900 kg/m^3^ density foamed concrete respectively.

**Table 1:** Percentage of 700 kg/m^3^ density foamed concrete compressive strength retained at predetermined exposed temperature.

Specimen	Exposed temperature (°C)					
	20 °C	105 °C	200 °C	400 °C	600 °C	800 °C
Control	100%	98%	91%	73%	40%	0%
0.1CNF	100%	98%	92%	82%	54%	15%
0.2CNF	100%	98%	94%	83%	57%	18%
0.3CNF	100%	99%	94%	83%	58%	22%
0.4CNF	100%	98%	93%	81%	57%	17%
0.5CNF	100%	98%	94%	80%	56%	16%
0.6CNF	100%	98%	92%	79%	52%	14%

**Table 2:** Percentage of 1100 kg/m^3^ density lightweight foamed concrete compressive strength retained at predetermined exposed temperature.

Specimen	Exposed temperature (°C)					
	20 °C	105 °C	200 °C	400 °C	600 °C	800 °C
Control	100%	98%	91%	73%	43%	4%
0.1CNF	100%	99%	95%	82%	54%	17%
0.2CNF	100%	99%	95%	83%	57%	19%
0.3CNF	100%	98%	93%	81%	57%	24%
0.4CNF	100%	99%	95%	82%	60%	26%
0.5CNF	100%	98%	94%	80%	56%	22%
0.6CNF	100%	98%	93%	79%	52%	13%

**Table 3:** Percentage of 1500 kg/m^3^ density foamed concrete compressive strength retained at predetermined exposed temperature.

Specimen	Exposed temperature (°C)					
	20 °C	105 °C	200 °C	400 °C	600 °C	800 °C
Control	100%	98%	92%	73%	45%	7%
0.1CNF	100%	99%	95%	81%	54%	18%
0.2CNF	100%	99%	95%	82%	55%	20%
0.3CNF	100%	98%	93%	81%	57%	22%
0.4CNF	100%	98%	94%	81%	59%	24%
0.5CNF	100%	99%	96%	83%	61%	26%
0.6CNF	100%	98%	93%	79%	54%	21%

**Table 4:** Percentage of 1900 kg/m^3^ density foamed concrete compressive strength retained at predetermined exposed temperature.

Specimen	Exposed temperature (°C)					
	20 °C	105 °C	200 °C	400 °C	600 °C	800 °C
Control	100%	98%	92%	73%	45%	9%
0.1CNF	100%	99%	95%	81%	54%	19%
0.2CNF	100%	99%	95%	81%	55%	21%
0.3CNF	100%	98%	93%	81%	57%	24%
0.4CNF	100%	98%	94%	81%	59%	25%
0.5CNF	100%	98%	95%	83%	63%	26%
0.6CNF	100%	98%	93%	79%	54%	22%

**Table 5:** Percentage of 700 kg/m^3^ density lightweight foamed concrete flexural strength retained at predetermined exposed temperature.

Specimen	Exposed temperature (°C)					
	20 °C	105 °C	200 °C	400 °C	600 °C	800 °C
Control	100%	99%	90%	71%	39%	0%
0.1CNF	100%	99%	93%	84%	52%	15%
0.2CNF	100%	98%	93%	84%	54%	18%
0.3CNF	100%	99%	95%	85%	59%	25%
0.4CNF	100%	98%	92%	79%	58%	20%
0.5CNF	100%	98%	95%	82%	59%	16%
0.6CNF	100%	99%	94%	81%	50%	14%

**Table 6:** Percentage of 1100 kg/m^3^ density foamed concrete flexural strength retained at predetermined exposed temperature.

Specimen	Exposed temperature (°C)					
	20 °C	105 °C	200 °C	400 °C	600 °C	800 °C
Control	100%	96%	87%	69%	37%	4%
0.1CNF	100%	97%	91%	79%	52%	15%
0.2CNF	100%	98%	93%	82%	54%	18%
0.3CNF	100%	97%	92%	81%	59%	25%
0.4CNF	100%	99%	95%	79%	61%	28%
0.5CNF	100%	97%	91%	82%	59%	20%
0.6CNF	100%	98%	93%	79%	54%	14%

**Table 7:** Percentage of 1500 kg/m^3^ density lightweight foamed concrete flexural strength retained at predetermined exposed temperature.

Specimen	Exposed temperature (°C)					
	20 °C	105 °C	200 °C	400 °C	600 °C	800 °C
Control	100%	95%	87%	70%	39%	8%
0.1CNF	100%	98%	93%	82%	56%	22%
0.2CNF	100%	98%	93%	81%	54%	18%
0.3CNF	100%	98%	94%	77%	56%	25%
0.4CNF	100%	99%	95%	79%	61%	26%
0.5CNF	100%	99%	95%	84%	63%	29%
0.6CNF	100%	98%	93%	74%	51%	17%

**Table 8:** Percentage of 1900 kg/m^3^ density lightweight foamed concrete flexural strength retained at predetermined exposed temperature.

Specimen	Exposed temperature (°C)					
	20 °C	105 °C	200 °C	400 °C	600 °C	800 °C
Control	100%	95%	87%	70%	39%	9%
0.1CNF	100%	98%	93%	82%	56%	24%
0.2CNF	100%	98%	93%	81%	54%	21%
0.3CNF	100%	99%	95%	77%	56%	26%
0.4CNF	100%	100%	96%	80%	62%	28%
0.5CNF	100%	98%	94%	83%	62%	29%
0.6CNF	100%	97%	92%	73%	50%	17%

## 2 Experimental design, materials, and methods

The research program started with mix design process and formulation of foamed concrete with the addition of coir fibre (by volume fraction). Moreover, it should be noted that the foamed concrete mix proportions (sand: cement: water) was fixed at the ratio of 1:1.5:0.45. Meanwhile, several mixtures were also prepared with different percentages of *coir fibre* as follows: 0.1%, 0.2%, 0.3%, 0.4%, 0.5%, and 0.6% by mix volume. Next, four different densities of 700, 1100, 1500 and 1900 kg/m^3^ were prepared. Small variations in the densities will only produce small values in the properties; thus these four densities were opted in this study to have comparable results for a better understanding of the properties when exposed to elevated temperatures. The 700 kg/m^3^ density can be considered for non-structural application, 1100 and 1500 kg/m^3^ densities for semi-structural application and 1900 kg/m^3^ density for structural purpose.

The next step involved the production of the foamed concrete specimens in substantial quantities which was put through curing process, followed by the exposure to oven drying with the aim of achieving the target densities. Following this process, the specimens underwent moisture curing for specific days (sealed cured with plastic sheets). Elevated temperature test was conducted at day-28.

**Fig. 1:**
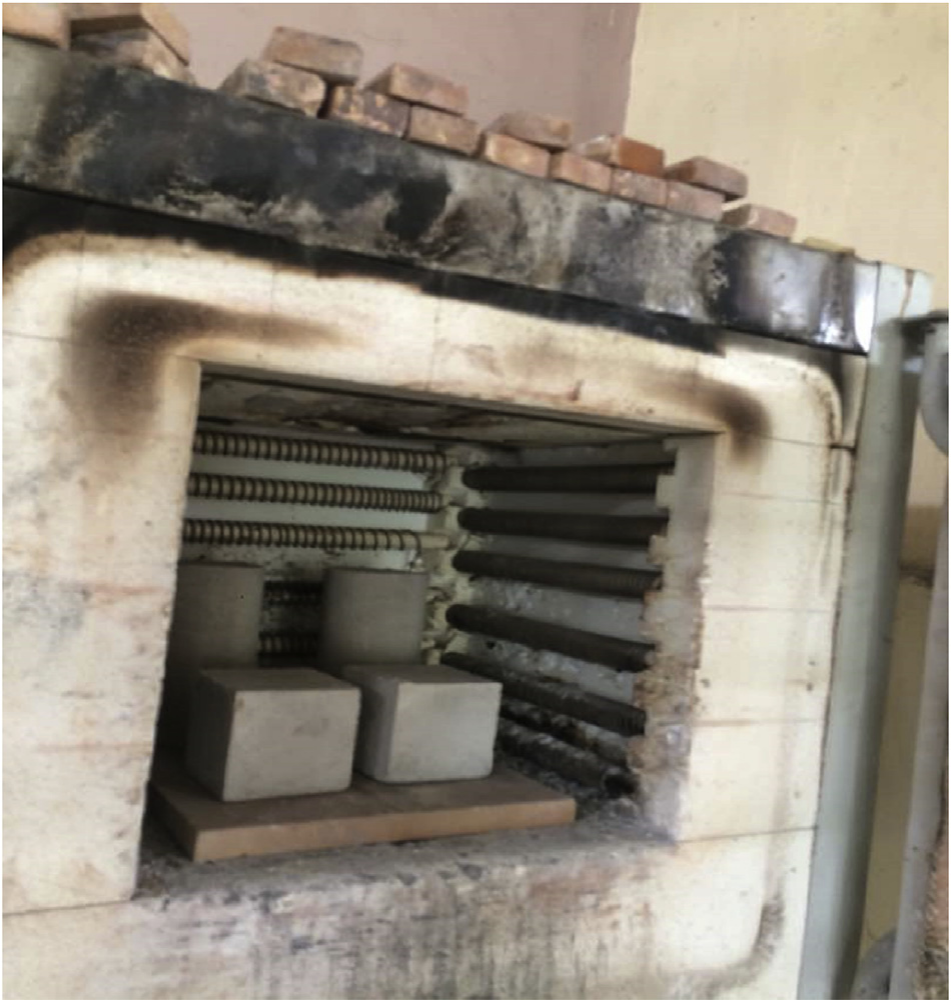
Foamed concrete specimens were exposed to elevated temperatures.

Electric furnace was used for heating the LFC specimens to the various steady-state temperatures. Heating temperatures were set at 105 °C, 200 °C, 300 °C, 400 °C, 500 °C, 600 °C, 700 °C and 800 °C. The electric furnace temperature exposure profiles were produced by a programmable microprocessor temperature controller attached to the furnace power supply and monitored by a Type K thermocouple located in the furnace chamber. The high temperature furnace (Fig. 1) had a maximum operating temperature of 1200 °C. Pre-testing checking of the furnace showed that the furnace controllers and furnace power system could maintain furnace operating temperatures within ±1 °C over the test range.

**Fig. 2:**
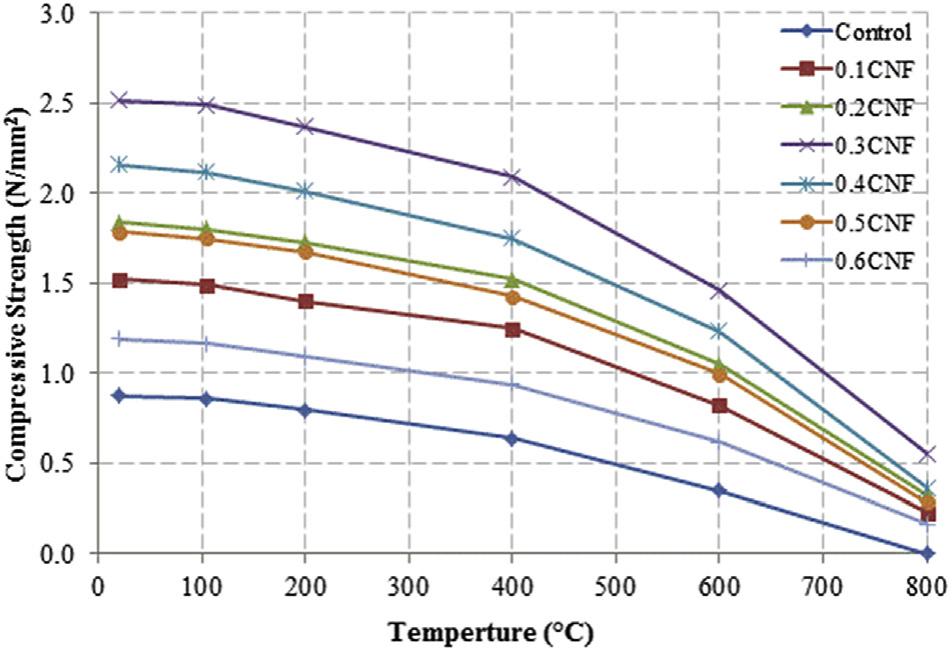
Compressive strength of 700 kg/m^3^ density foamed concrete as a function of temperature.

**Fig. 3:**
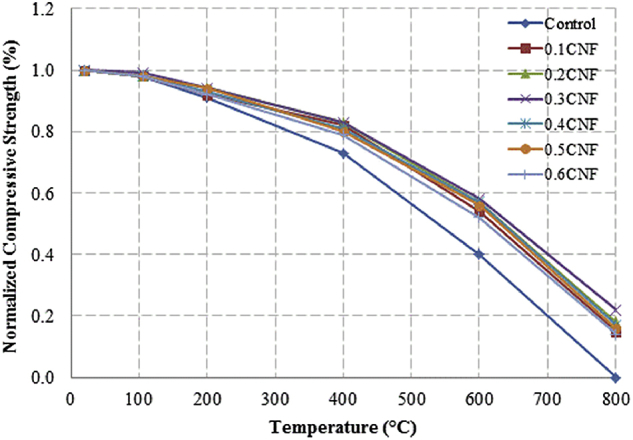
Normalized compressive strength of 700 kg/m^3^ density foamed concrete as a function of temperature.

**Fig. 4:**
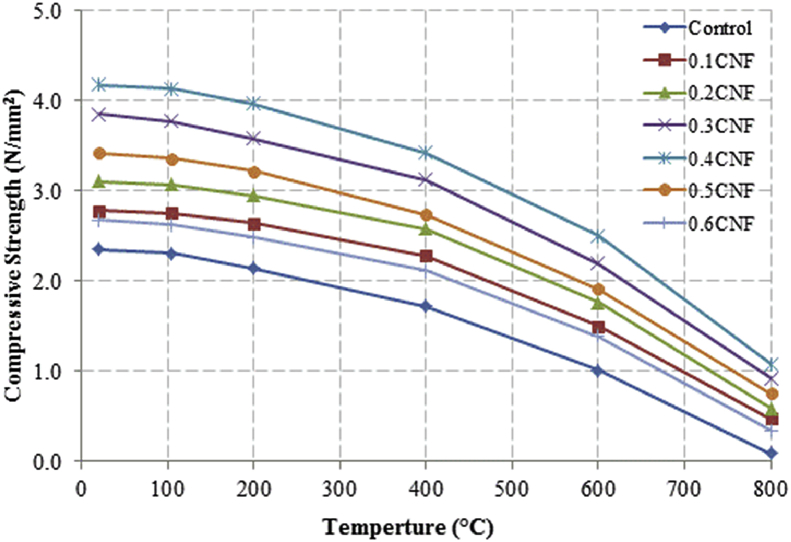
Compressive strength of 1100 kg/m^3^ density foamed concrete as a function of temperature.

**Fig. 5:**
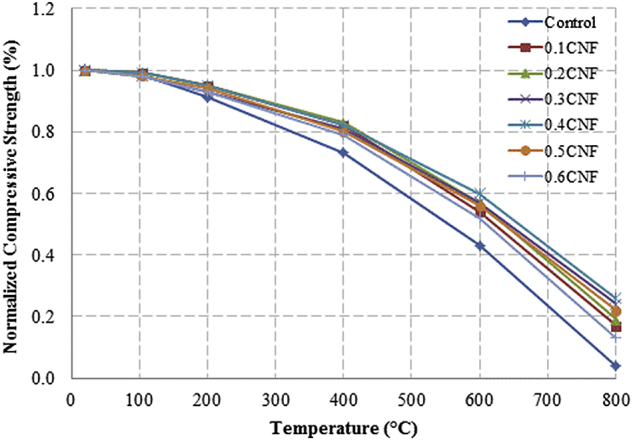
Normalized compressive strength of 1100 kg/m^3^ density lightweight foamed concrete as a function of temperature.

**Fig. 6:**
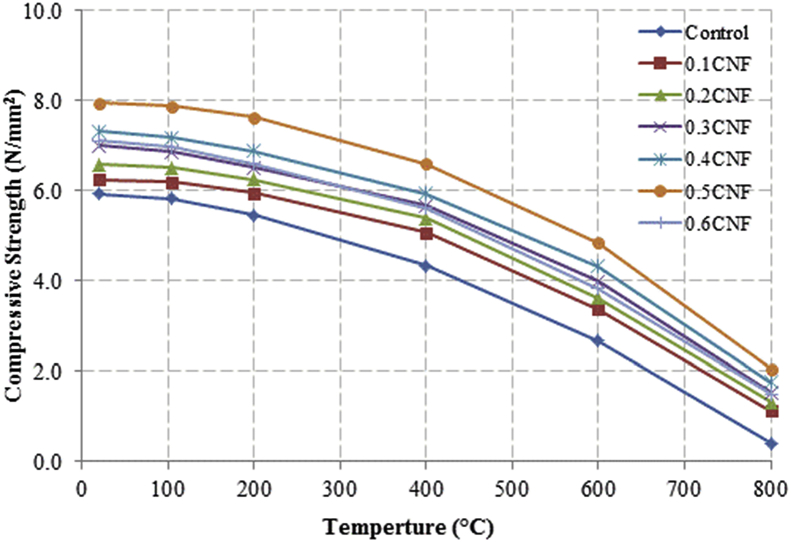
Compressive strength of 1500 kg/m^3^ density foamed concrete as a function of temperature.

**Fig. 7:**
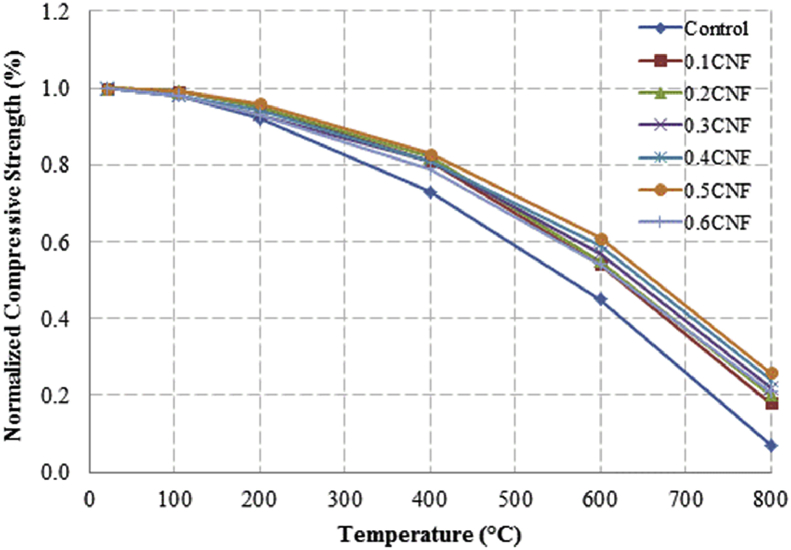
Normalized compressive strength of 1500 kg/m^3^ density foamed concrete as a function of temperature.

**Fig. 8:**
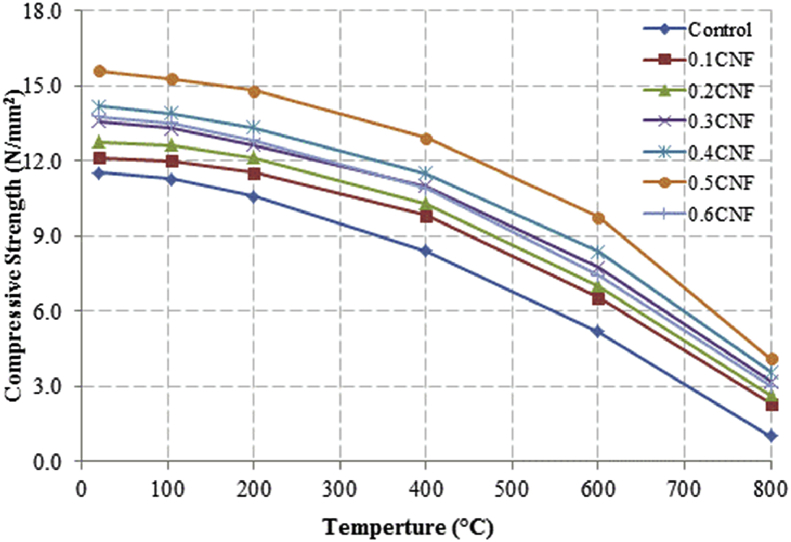
Compressive strength of 1900 kg/m^3^ density foamed concrete as a function of temperature.

**Fig. 9:**
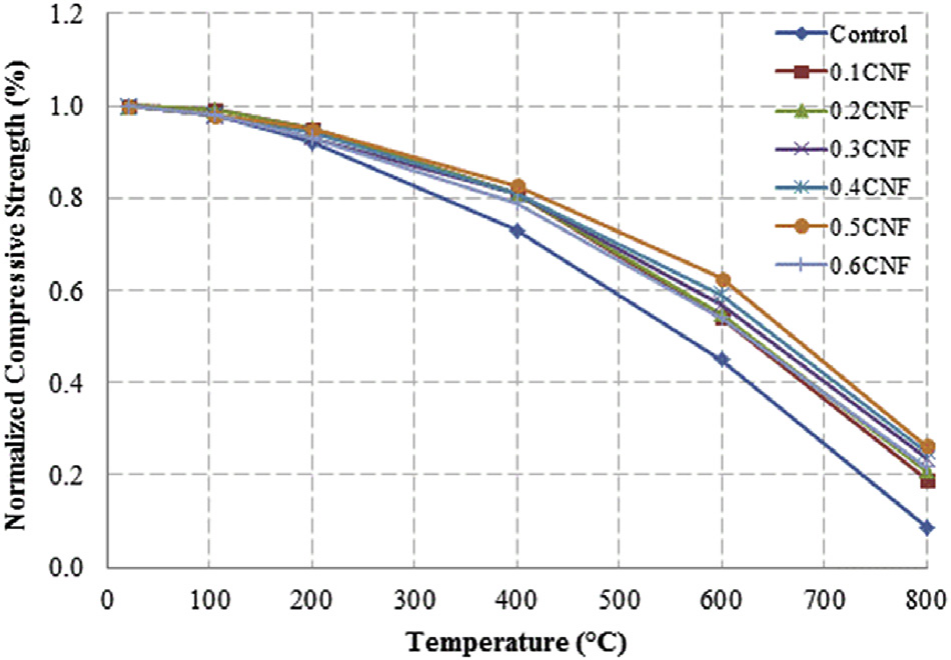
Normalized compressive strength of 1900 kg/m^3^ density foamed concrete as a function of temperature.

**Fig. 10:**
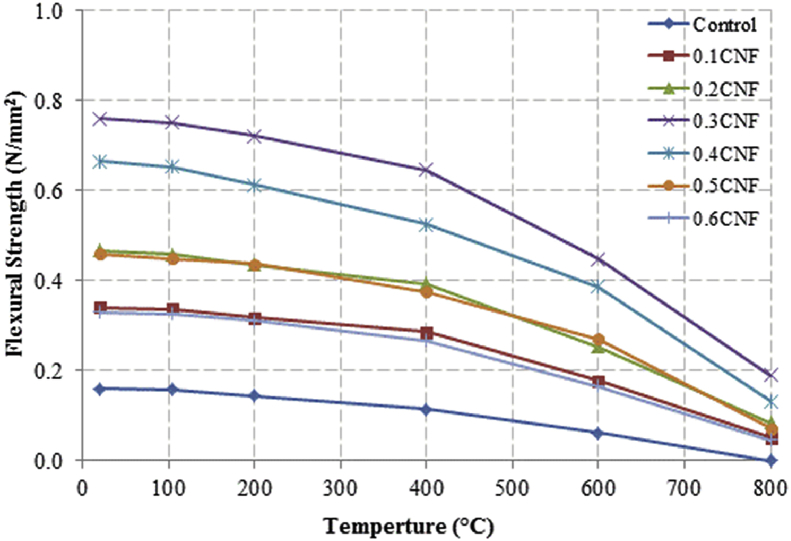
Flexural strength of 700 kg/m^3^ density foamed concrete as a function of temperature.

**Fig. 11:**
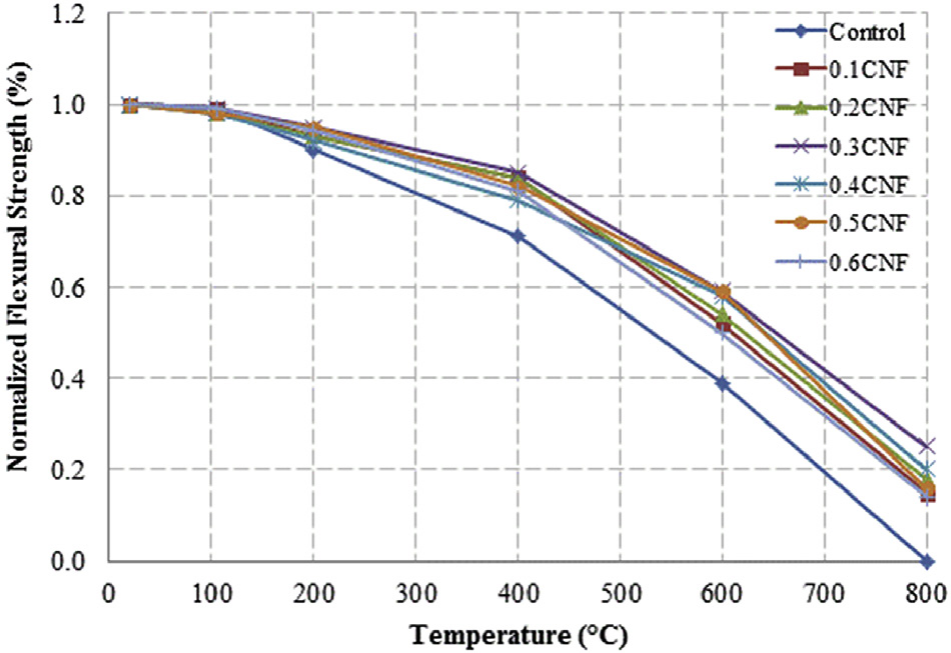
Normalized flexural strength of 700 kg/m^3^ density lightweight foamed concrete as a function of temperature.

**Fig. 12:**
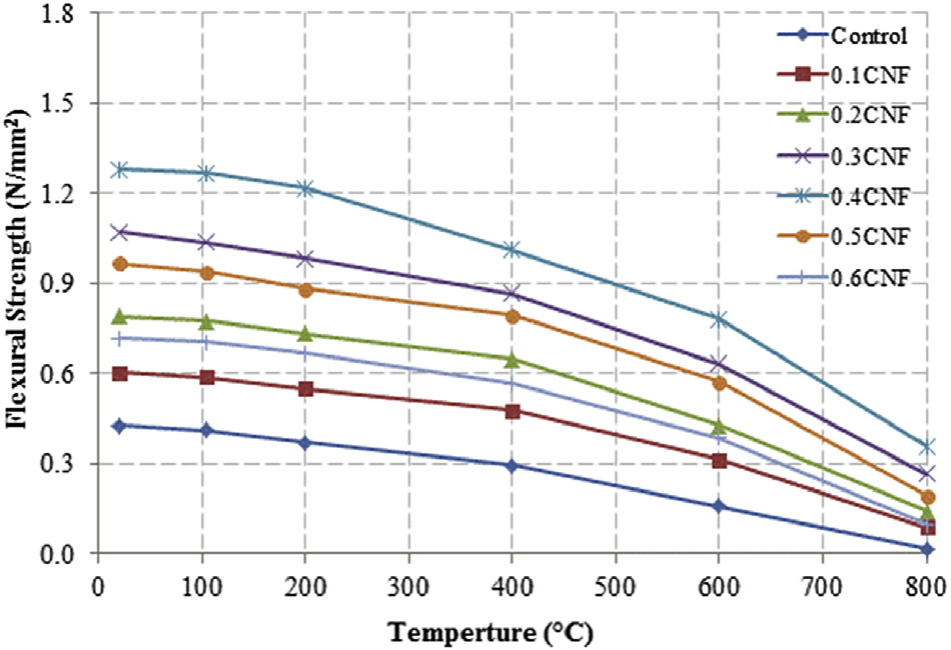
Flexural strength of 1100 kg/m^3^ density foamed concrete as a function of temperature.

**Fig. 13:**
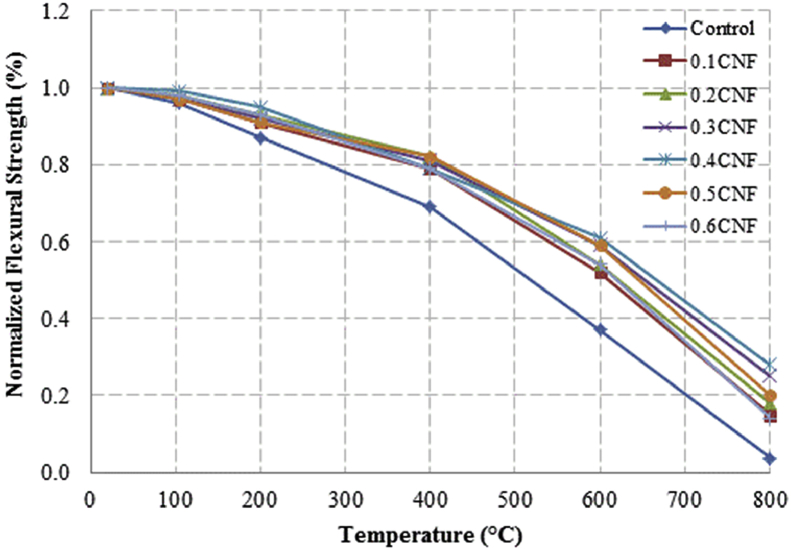
Normalized flexural strength of 1100 kg/m^3^ density foamed concrete as a function of temperature.

**Fig. 14:**
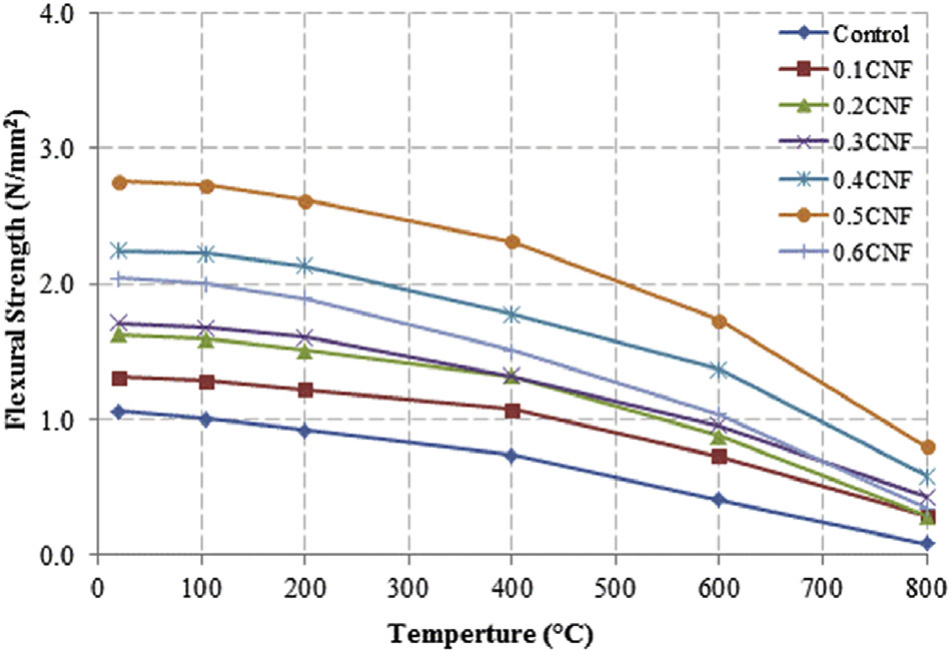
Flexural strength of 1500 kg/m^3^ density foamed concrete as a function of temperature.

**Fig. 15:**
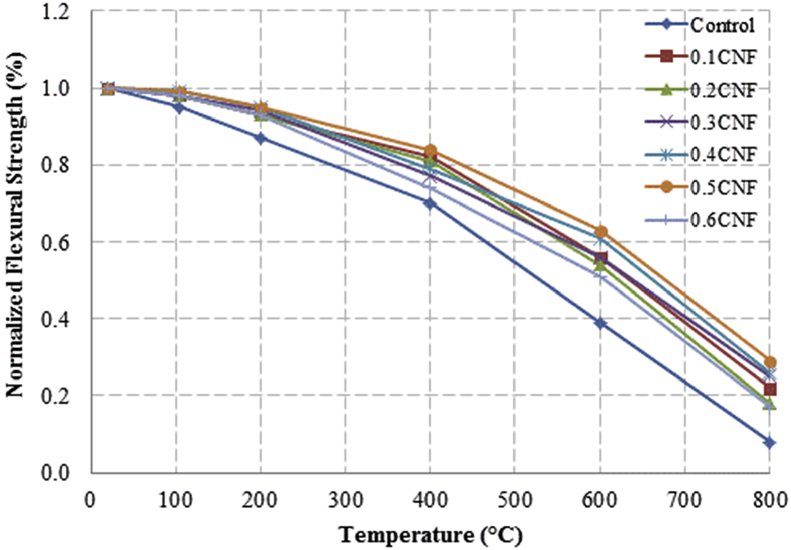
Normalized flexural strength of 1500 kg/m^3^ density lightweight foamed concrete as a function of temperature.

**Fig. 16:**
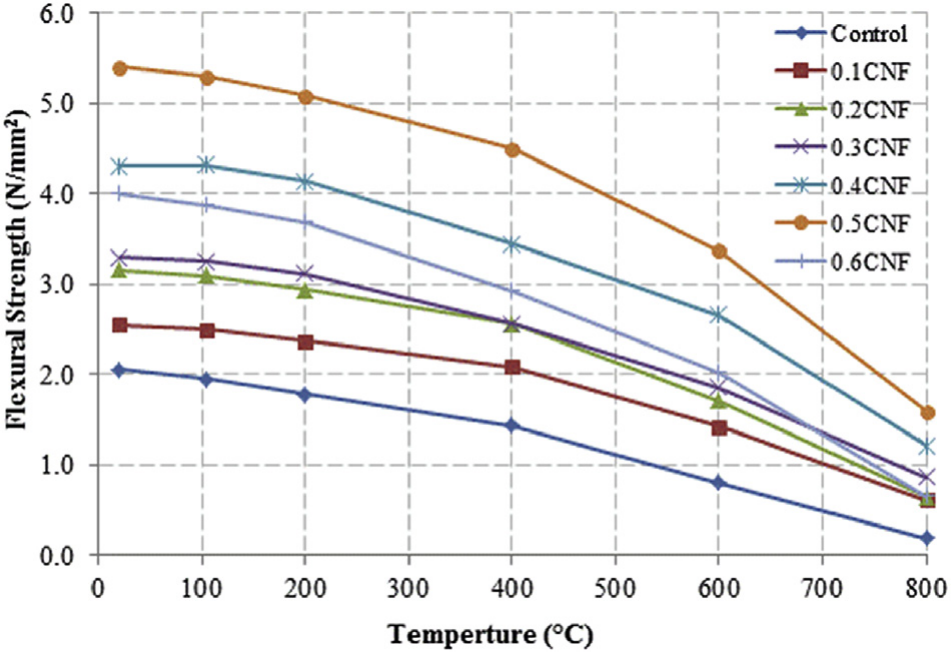
Flexural strength of 1900 kg/m^3^ density foamed concrete as a function of temperature.

**Fig. 17:**
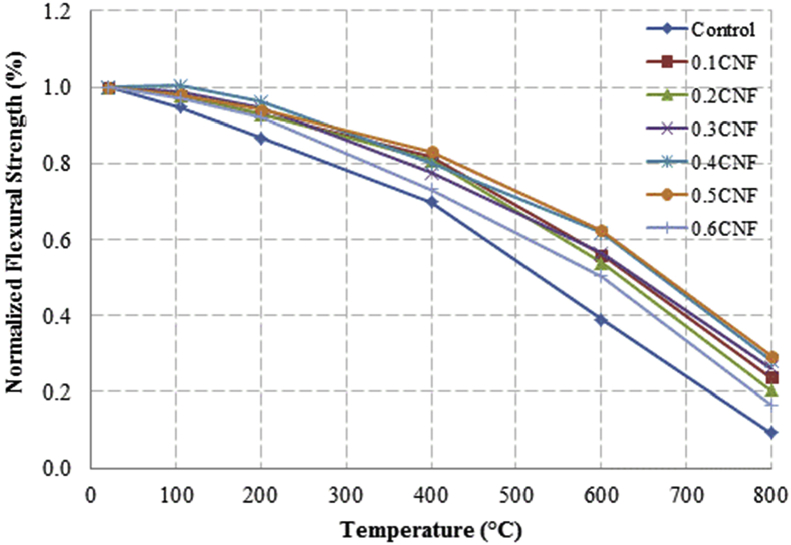
Normalized flexural strength of 1900 kg/m^3^ density lightweight foamed concrete as a function of temperature.

The elevated temperature compressive strength test was conducted using universal testing machine with the capacity of 3000KN. In the context of the present study, the test was performed in accordance with BS EN 12390-3:2009 [1] using a cubic shaped specimen with the size of 100 mm × 100 mm x 100mm. The loading rate for compression and flexural tests was set at 0.2 N/sec.

For elevated temperature flexural strength test, rectangular beam of 100mmx100 mm × 500 mm was employed, while the two points loading arrangement was utilized according to the method BS 1521:1997 [2]. The nominal distance between the supports was 300mmIn addition, the rollers enabled free horizontal movement, while the samples of foamed concrete specimens were applied at a constant displacement rate of 0.2 N/sec based on the optimum value determined experimentally.

Loading was applied after removing the test specimens from the electric furnace. Each specimen was wrapped with insulation sheets immediately after being removed from the electric furnace to minimize heat loss from the specimen to atmosphere. For each set, three replicate tests were carried out to check consistency of results. During the loading process, the temperature of each sample was measured and it was found that the temperature was stable throughout the testing period.
